# Application of functional vincristine plus dasatinib liposomes to deletion of vasculogenic mimicry channels in triple-negative breast cancer

**DOI:** 10.18632/oncotarget.5382

**Published:** 2015-09-28

**Authors:** Fan Zeng, Rui-Jun Ju, Lei Liu, Hong-Jun Xie, Li-Min Mu, Yao Zhao, Yan Yan, Ying-Jie Hu, Jia-Shuan Wu, Wan-Liang Lu

**Affiliations:** ^1^ Beijing Key Laboratory of Molecular Pharmaceutics and New Drug System, State Key Laboratory of Natural and Biomimetic Drugs, School of Pharmaceutical Sciences, Peking University, Beijing 100191, China

**Keywords:** functional liposome, vincristine, dasatinib, vasculogenic mimicry channel, triple-negative breast cancer

## Abstract

Standard chemotherapy cannot eradicate triple-negative breast cancer (TNBC) while the residual cancer cells readily form the vasculogenic mimicry (VM) channels, which lead to the relapse of cancer after treatment. In this study, the functional vincristine plus dasatinib liposomes, modified by a targeting molecule DSPE-PEG_2000_-c(RGDyK), were fabricated to address this issue. The investigations were performed on TNBC MDA-MB-231 cells and MDA-MB-231 xenografts in nude mice. The liposomes exhibited the superior performances in the following aspects: the enhancement of cellular uptake via targeted action; the induction of apoptosis via activation of caspase 8, 9, and 3, increased expression of Bax, decreased expression of Mcl-1, and generation of reactive oxygen species (ROS); and the deletion of VM channels via inhibitions on the VM channel indicators, which consisted of vascular endothelial-cadherin (VE-Cad), focal adhesion kinase (FAK), phosphatidylinositide 3-kinase (PI3K), and matrix metallopeptidases (MMP-2, and MMP-9). Furthermore, the liposomes displayed the prolonged circulation time in the blood, the increased accumulation in tumor tissue, and the improved therapeutic efficacy along with deletion of VM channels in the TNBC-bearing mice. In conclusion, the nanostructured functional drug-loaded liposomes may provide a promising strategy for the treatment of invasive TNBC along with deletion of VM channels.

## INTRODUCTION

Breast cancer is the most common malignant disease and a major cause of mortality among women worldwide [1]. Nearly 1.4 million individuals are diagnosed with breast cancer globally, with more than 450,000 deaths per year [2]. Among these cases, approximately 15–20% are characterized as the triple-negative breast cancer (TNBC) phenotype, namely, the absence of estrogen receptors, progesterone receptors and human epidermal growth factor receptor 2 [3, 4]. Patients with TNBC have a very poor prognosis because TNBC is highly invasive and associated with a high rate of cancer metastasis and recurrence [5, 6].

In recent years, increasing evidences have indicated that highly patterned vasculogenic mimicry (VM) channels are formed by TNBC cells instead of endothelial cells as an alternative microcirculation pathway, and these channel-forming TNBC cells are highly drug resistant [7, 8]. Comprehensive treatments, which consist of surgery, radiation and standard chemotherapy, cannot completely eliminate TNBC cells [9, 10]. In hypoxic conditions, the residual cancer cells readily proliferate via the formation of highly patterned VM channels, which provide nutrients to the relapsed cancer cells. Consequently, the elimination of VM channels plays a crucial role in a successful treatment of TNBC.

In this study, we hypothesized that functional vincristine plus dasatinib liposomes could eliminate TNBC cells along with destroying VM channels. In these nanostructured liposomes, a cyclic peptide c(RGD_y_K) was conjugated with N-hydroxysuccinimidyl-polyethylene glycol distearoylphosphatidyl ethanolamine (DSPE-PEG_2000_-NHS) and used as a targeting molecule to modify the liposome surface. Vincristine was used as an anticancer drug, and dasatinib was employed as VM channel inhibitor.

Vincristine is a cell cycle-specific anticancer agent. The cytotoxic activity of vincristine is related to its activities regarding the inhibition of microtubules and the alteration of tubulin polymerization equilibrium, which thus causes the arrest of cell division in metaphase [11]. Dasatinib is an inhibitor of sarcoma gene family kinases (SFKs) [12]. Studies indicate that SFKs play a central role in multiple signaling pathways that regulate cell adhesion, invasion and motility, and are also involved in interactions with numerous breast cancer associated growth factors [13, 14]. Thus, as an inhibitor of SFKs, dasatinib exhibits multiple effects on cancer, including anti-proliferative activity, induction of apoptosis, and inhibition of invasion [15–17]. Because of these properties, dasatinib represents a promising inhibitor in TNBC treatment.

Conventional chemotherapy has demonstrated unfavorable pharmacokinetic properties and systemic toxicities as a result of the direct delivery of free drugs into the circulatory system [18]. Pegylated liposomes with suitable particle sizes have been demonstrated to improve the therapeutic index of drugs by prolonged circulation in the blood and enhanced tumor accumulation in cancer tissue due to the enhanced permeability and retention (EPR) effects [19, 20]. Besides, the liposomes are biocompatible and can entrap both hydrophilic and hydrophobic pharmaceutical agents, protecting them from external damage and allowing to local concentrated drug delivery. Therefore, development of a tool to allow for constant and selective delivery of therapeutics is desirable. Active targeting was achieved by conjugating c(RGD_y_K) with DSPE-PEG_2000_-NHS and modifying the liposome surface in this study. C(RGD_y_K) is a cyclic peptide that has a specific affinity for the integrin receptor, which is overexpressed on many malignant cancer cells [21, 22]. This cyclic peptide offers a potential avenue for nanostructured drug carriers to access target cancer cells.

**Figure 1:**
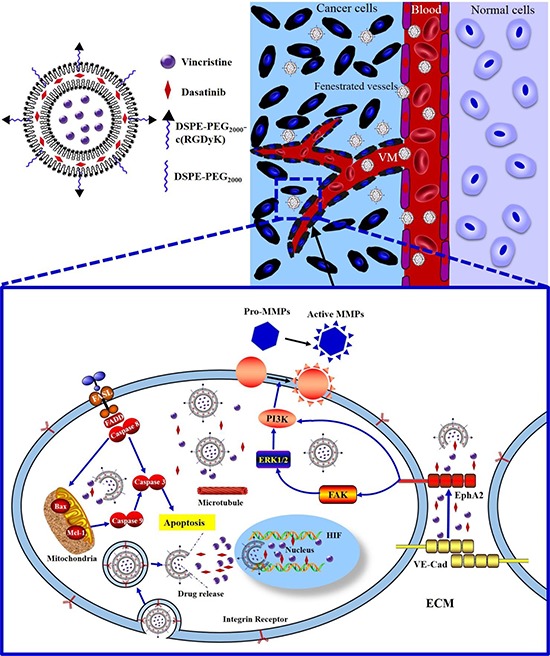
Schematic design and mechanism of functional vincristine plus dasatinib liposomes for the treatment of TNBC and VM channel elimination Notes: During hypoxic conditions, the transcription of VE-Cad is increased, and EphA2 is subsequently re-localized to the cell membrane and phosphorylated. Phosphorylated EphA2 directly activates PI3K or initiates the activation of FAK and downstream PI3K. Activated PI3K, in turn, activates MMPs (MMP-2 and MMP-9), which eventually results in VM channel formation. Functional vincristine plus dasatinib liposomes specifically bind to integrin receptors on cancer cells. The internalized liposomes induce the apoptosis of cancer cells through a cascade of apoptotic reactions via the activation of caspase 8, 9, and 3, the increased expression of the pro-apoptotic protein Bax, the decreased expression of anti-apoptotic protein Mcl-1, and generation of ROS. Furthermore, they destroy VM channels via the decreased expression of VE-Cad, FAK, PI3K, MMP-2 and MMP-9.

The objectives of this study were to fabricate functional vincristine plus dasatinib liposomes to eliminate VM channels in the treatment of invasive TNBC and to define the action mechanism (Figure 1).

## RESULTS

### Fabrication of functional vincristine plus dasatinib liposomes

**Figure 2:**
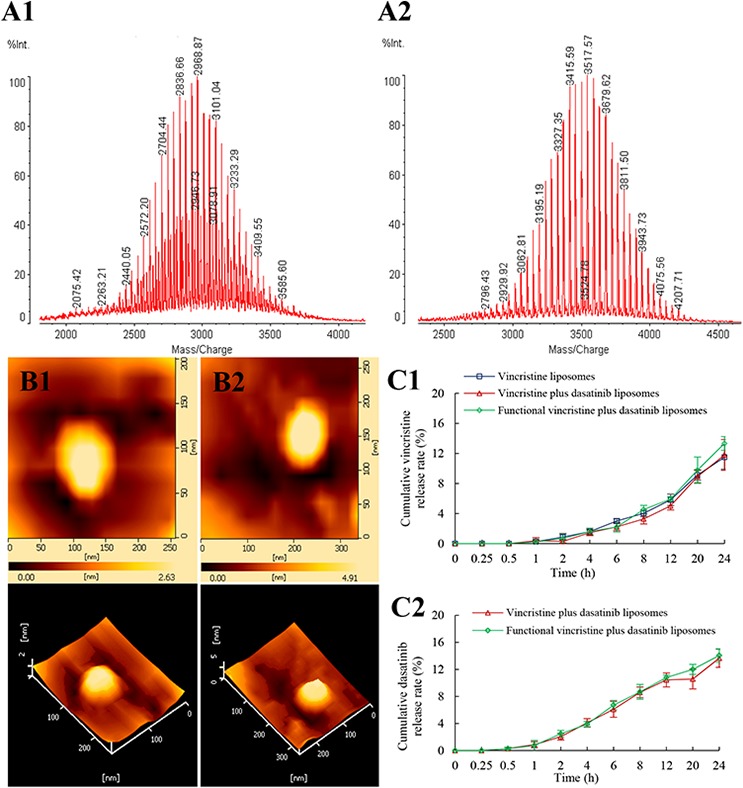
Characterization of targeting molecules and functional vincristine plus dasatinib liposomes Notes: MALD-TOF-MS spectra of (A1) DSPE-PEG_2000_-NHS and (A2) DSPE-PEG_2000_-c(RGDyK) targeting molecule. AFM images of (B1) vincristine liposomes and (B2) functional vincristine plus dasatinib liposomes. Release rates of (C1) vincristine and (C2) dasatinib from the liposomes. Data are presented as the mean ± SD (*n* = 3).

The characterization of targeting molecule and liposomes were shown in Figure 2. The results from the matrix-assisted laser desorption/ionization time of flight mass spectrometer (MALDI-TOF-MS) spectra of DSPE-PEG_2000_-NHS (Figure 2A1) and DSPE-PEG_2000_-c(RGDyK) (Figure 2A2) indicated that the c(RGDyK) peptide was successfully conjugated to the distal end of DSPE-PEG_2000_-NHS via a nucleophilic substitution reaction. Observation under an atomic force microscope (AFM) indicated that the liposome was round in shape with a smooth surface and approximately 100 nm in diameter (Figure 2B1 and 2B2). The *in vitro* release rates of vincristine and dasatinib from the liposomes were < 3% within the initial 2 h and were < 20% over 24 h (Figure 2C).

Table 1 lists the characterization of functional drugs-loaded liposomes. The results indicated that the average particle sizes of the liposomes were in the range of 100–107 nm with a narrow polydispersity index (PDI; ≤ 0.2), and the charge values were slightly negative (−6 mV). The encapsulation efficiencies of vincristine and dasatinib were both > 90% in all prepared liposomes.

**Table 1:** Characterization of the liposomes

Liposomes	Particle size (nm)	Polydispersity Index	Zeta potential (mV)	Encapsulation efficiency (%)	
				vincristine	dasatinib
Blank functional liposomes	103.10 ± 1.54	0.17 ± 0.01	−5.48 ± 0.35	-	-
Vincristine liposomes	103.37 ± 2.27	0.17 ± 0.01	−8.61 ± 0.83	97.92 ± 0.85	-
Vincristine plus dasatinib liposomes	104.73 ± 2.02	0.16 ± 0.03	−8.57 ± 0.09	98.21 ± 0.78	93.29 ± 0.75
Functional vincristine plus dasatinib liposomes	104.50 ± 1.97	0.17 ± 0.02	−6.21 ± 0.41	97.72 ± 0.87	91.79 ± 0.42

Data are presented as the mean ± SD (*n* = 3).

### Cellular uptake by TNBC cells and targeting effect

**Figure 3:**
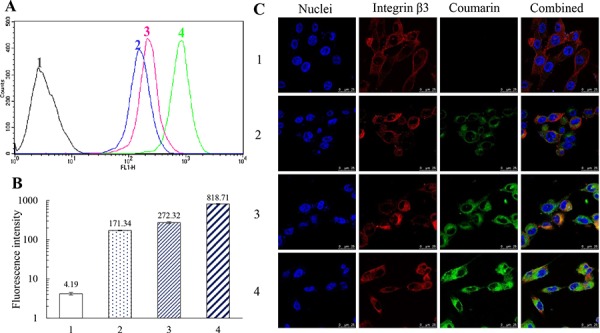
Cellular uptake by MDA-MB-231 cells and targeting effect of functional coumarin liposomes Notes: **A, B.** Evaluation of cellular uptake via flow cytometry. Data are presented as the mean ± SD (*n* = 3). **C.** Observation of targeting effect under a confocal microscope (scale bar = 25 μm). 1, blank control; 2, coumarin liposomes; 3, functional coumarin liposomes; 4, free coumarin.

To evaluate the cellular uptake by TNBC cells and targeting effect, the fluorescence probe coumarin was used to label the liposomes. Figure 3A and 3B indicate the cellular uptake by MDA-MB-231 cells after treatments with varying formulations. Results showed that the rank of cellular uptake was free coumarin > functional coumarin liposomes > coumarin liposomes > blank control.

Figure 3C displays the targeting effect of varying coumarin labeled liposomes. In the confocal images, integrin β3 receptor exhibited red fluorescence, whereas the nuclei were stained in blue. Bright yellow fluorescence was a composite image of green and red fluorescence, and used to indicate the targeting effect of the liposomes with integrin β3 receptor on MDA-MB-231 cells. Results demonstrated that the functional coumarin liposomes were bound with the integrin β3 receptor and exhibited substantially higher green fluorescence intensity in the MDA-MB-231 cells compared with the coumarin liposomes, which suggests that more functional liposomes had been internalized by the cancer cells.

### Inhibitory effects and induction of apoptosis on TNBC cells

**Figure 4:**
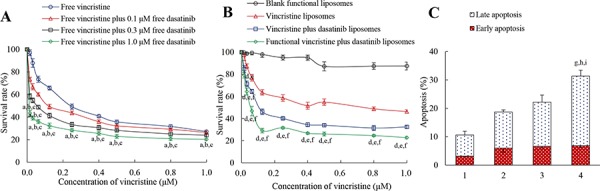
Inhibitory effect and induced apoptosis of MDA-MB-231 cells after treatment with functional vincristine plus dasatinib liposomes Notes: Inhibitory effects on MDA-MB-231 cells after treatments with **A.** free drugs and **B.** varying liposomes for 48 h. *p* < 0.05, a, vs. free vincristine; b, vs. free vincristine plus 0.1 μM free dasatinib; c, vs. free vincristine plus 0.3 μM free dasatinib; d, vs. blank functional liposomes; e, vs. vincristine liposomes; f, vs. vincristine plus dasatinib liposomes. Data are presented as the mean ± SD (*n* = 6). **C.** Induced apoptotic percentages of MDA-MB-231 cells. Data are presented as the mean ± SD (*n* = 3). 1, control; 2, vincristine liposomes; 3, vincristine plus dasatinib liposomes; 4, functional vincristine plus dasatinib liposomes. *p* < 0.05, g, vs. 1; h, vs. 2; i, vs. 3.

Figure 4A illustrates the inhibitory effects of free drugs on MDA-MB-231 cells. Results demonstrated that free vincristine alone had limited efficacy in inhibiting MDA-MB-231 cells; in contrast, in the co-treatment of free vincristine with free dasatinib, the inhibitory effect of vincristine on the cancer cells was significantly enhanced in a dasatinib concentration-dependent manner. Figure 4B demonstrates the inhibitory effects of functional vincristine plus dasatinib liposomes. Among the four types of liposomes, functional vincristine plus dasatinib liposomes exhibited the strongest inhibitory effects on cancer cells at various dose levels. The blank functional liposomes exhibited a minimal cytotoxic effect toward cancer cells.

Figure 4C depicts the induced apoptosis on MDA-MB-231 cells. After incubation with blank medium, vincristine liposomes, vincristine plus dasatinib liposomes and functional vincristine plus dasatinib liposomes, the total percentages of apoptosis were 10.60 ± 1.20, 18.70 ± 0.78, 22.16 ± 2.32 and 31.36 ± 1.51%, respectively.

### Apoptotic signaling pathways of TNBC cells

**Figure 5:**
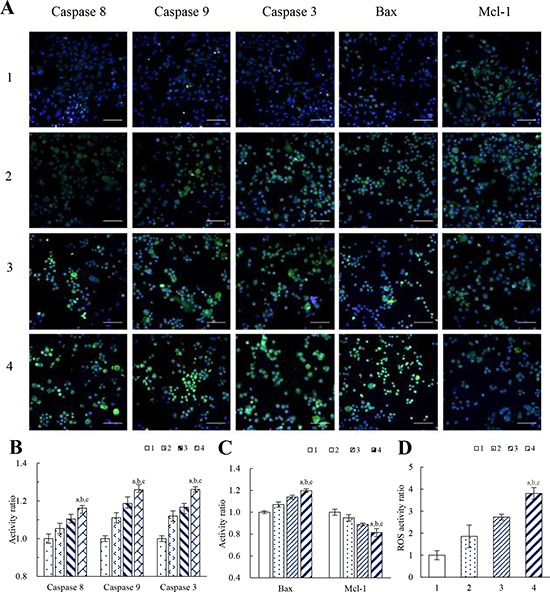
Effects on apoptotic enzymes, apoptotic proteins and ROS level of MDA-MB-231 cells after treatment with functional vincristine plus dasatinib liposomes Notes: **A.** Fluorescent images of the expressed apoptotic enzymes and apoptotic proteins (scale bar = 100 μm). **B.** Activity ratios of caspase 8, caspase 9 and caspase 3. **C.** Activity ratios of Bax and Mcl-1. Data are presented as the mean ± SD (*n* = 6). **D.** ROS activity ratio. Data are presented as the mean ± SD (*n* = 3). 1, blank control; 2, vincristine liposomes; 3, vincristine plus dasatinib liposomes; 4, functional vincristine plus dasatinib liposomes. *p* < 0.05, a, vs. 1; b, vs. 2; c, vs. 3.

Figure 5A displays the expression levels of representative apoptotic enzymes and related apoptotic proteins in cancer cells after treatments with varying formulations. As compared to the blank control, functional vincristine plus dasatinib liposomes significantly increased the expression levels of caspase 3, caspase 8, caspase 9, and pro-apoptotic protein Bax while significantly suppressed the expression level of anti-apoptotic protein Mcl-1.

Figure 5B and 5C display the corresponding quantification results of above apoptotic enzymes and proteins. After treatment with blank medium, vincristine liposomes, vincristine plus dasatinib liposomes and functional vincristine plus dasatinib liposomes, the activities of apoptotic enzymes caspase 8, 9, and 3, the pro-apoptotic protein Bax and the anti-apoptotic protein Mcl-1 were changed in varying discriminable degrees as follows: the activity ratios of caspase 8 were 1.00 ± 0.02, 1.05 ± 0.03, 1.10 ± 0.02 and 1.16 ± 0.03, respectively; the activity ratios of caspase 9 were 1.00 ± 0.02, 1.11 ±0.03, 1.19 ± 0.04 and 1.26 ± 0.01, respectively; and the activity ratios of caspase 3 were 1.00 ± 0.02, 1.12 ± 0.03, 1.17 ± 0.02 and 1.26 ± 0.02, respectively. The activity ratios of Bax were 1.00 ± 0.01, 1.07 ± 0.02, 1.14 ± 0.02 and 1.20 ± 0.02, respectively, and the activity ratios of Mcl-1 were 1.00 ± 0.03, 0.95 ± 0.03, 0.89 ± 0.01 and 0.81 ± 0.04, respectively.

Figure 5D shows the level of reactive oxygen species (ROS) after treatments with varying formulations. The rank of ROS levels after treatments was functional vincristine plus dasatinib liposomes > vincristine plus dasatinib liposomes > vincristine liposomes > blank medium.

### Destruction of VM channels of TNBC cells

**Figure 6:**
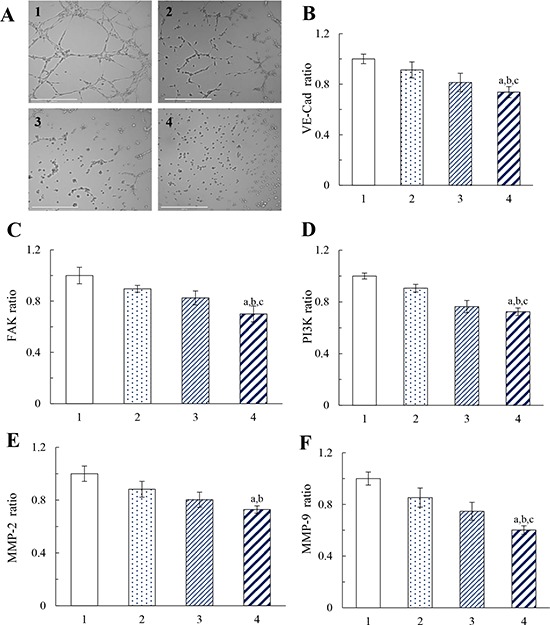
Destructive effects on the VM channels of MDA-MB-231 cells after treatment with functional vincristine plus dasatinib liposomes Notes: **A.** Destructive effects on the VM channels. Inhibitory effects on the VM channel indicators, including **B.** VE-Cad, **C.** FAK, **D.** PI3K, **E.** MMP-2, and **F.** MMP-9. Data are presented as the mean ± SD (*n* = 3). 1, blank control; 2, vincristine liposomes; 3, vincristine plus dasatinib liposomes; 4, functional vincristine plus dasatinib liposomes. *p* < 0.05, a, vs. 1; b, vs. 2; c, vs. 3.

Figure 6A displays the destructive effect on VM channels in a three dimensional matrigel culture model after treatments with varying formulations. The highly invasive MDA-MB-231 cells formed vessel-like loops, channels and networks after treatment with blank medium. The VM networks were significantly damaged after treatment with functional vincristine plus dasatinib liposomes, which exhibited the strongest destructive effect on the VM channels compared with other formulations.

Figure 6B to 6F illustrate the effects of varying formulations on the expressions of vascular endothelial-cadherin (VE-Cad), focal adhesion kinase (FAK), phosphatidylinositide 3-kinase (PI3K), and matrix metallopeptidases (MMP-2, and MMP-9) in MDA-MB-231 cells. After drug treatments, the inhibitory effects were evidenced by the expression ratios, which were ranked as follows: blank medium > vincristine liposomes > vincristine plus dasatinib liposomes > functional vincristine plus dasatinib liposomes. The functional vincristine plus dasatinib liposomes significantly inhibited the expression of VM indicators. The inhibition of these indicators for forming VM channels would facilitate the elimination of VM channels in TNBC treatment.

### Destruction of TNBC tumor spheroids

**Figure 7:**
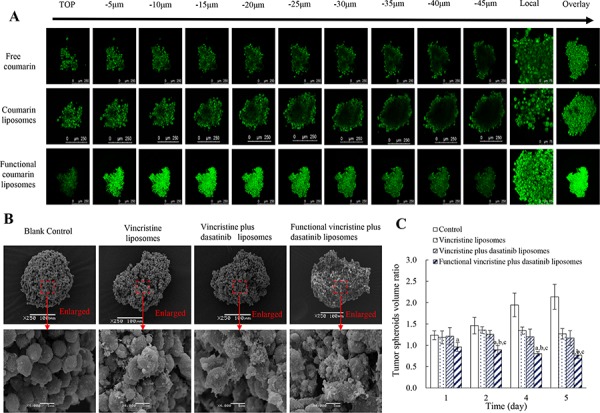
Penetrating ability and destructive effects on the tumor spheroids of MDA-MB-231 cells after treatment with functional liposomes Notes: **A.** Penetrative ability of functional liposomes to the spheroids (scale bar = 250 μm). **B.** Destructive effect on the spheroids (scale bar = 100 μm). **C.** Inhibitory effect on spheroid growth. Data are presented as the mean ± SD (*n* = 6). *p* < 0.05, a, vs. blank control; b, vs. vincristine liposomes; c, vs. vincristine plus dasatinib liposomes.

Figure 7A exhibits the penetration ability of coumarin-labeled liposomes into tumor spheroids. After incubation with varying formulations, images were taken in each layer of the spheroids. Results showed that the rank of fluorescent intensities in the spheroids was functional coumarin liposomes > free coumarin > coumarin liposomes, indicating that the functional liposomes had the strongest penetrating ability.

Figure 7B represents the destructive efficacy to tumor spheroids. After treatments with varying liposomes, functional vincristine plus dasatinib liposomes caused the most significant destructive effects, as the tightly organized spheroids were disintegrated.

Figure 7C displays the inhibitory effect on tumor spheroids. After treatment with blank medium, vincristine liposomes, vincristine plus dasatinib liposomes and functional vincristine plus dasatinib liposomes, the spheroid volume change ratios at day 5 were 2.14 ± 0.29, 1.27 ± 0.12, 1.17 ± 0.17 and 0.77 ± 0.08, respectively. Among these results, functional vincristine plus dasatinib liposomes exhibited the most significant inhibitory effects on tumor spheroid growth.

### Anticancer efficacy

**Figure 8:**
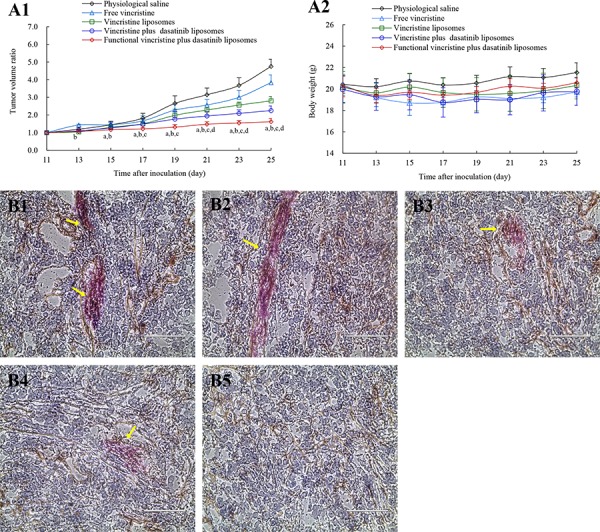

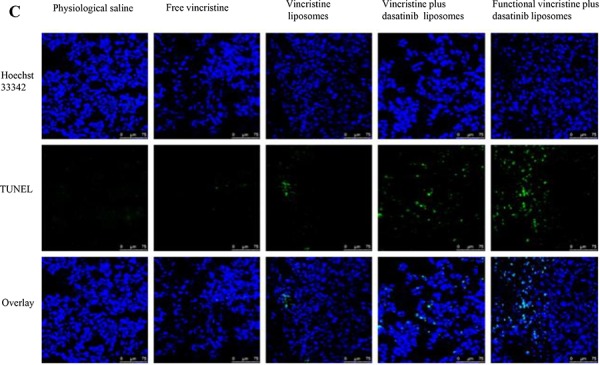

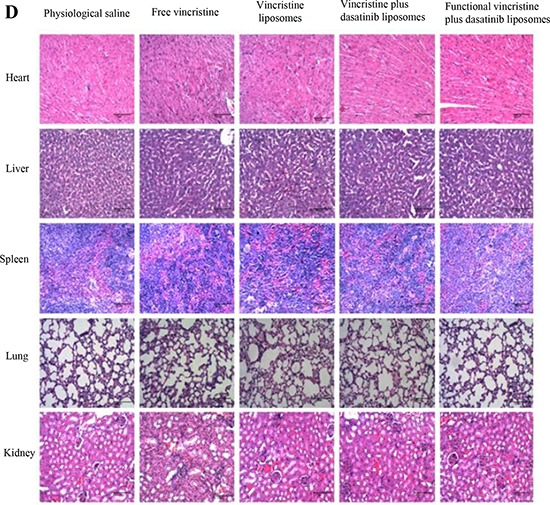
Antitumor efficacy in tumor-bearing nude mice xenografted with MDA-MB-231 cells after treatment with functional vincristine plus dasatinib liposomes Notes: Tumor volume ratio **A1.** and body weight changes **A2.** after treatments. Data are presented as the mean ± SD (*n* = 6). *p* < 0.05, a, vs. saline; b, vs. free vincristine; c, vs. vincristine liposomes; d, vincristine plus dasatinib liposomes. The destructive effect on the VM channels in nude mice after treatments with **B1.** physiological saline, **B2.** free vincristine, **B3.** vincristine liposomes, **B4.** vincristine plus dasatinib liposomes, and **B5.** functional vincristine plus dasatinib liposomes. **C.** TUNEL assay of the tumor tissues after treatments of varying formulations (scale bar = 75 μm). **D.** Histopathological observations of the heart, kidney, liver, lung, and spleen of tumor-bearing nude mice xenografted with MDA-MB-231 cells after treatments of varying formulations (scale bar = 100 μm).

Figure 8A1 indicates the anticancer efficacy in tumor-bearing mice xenografted with MDA-MB-231 cells. Using physiological saline as a blank control, the inhibitory ratios of the tumor volumes at day 25 were 19.31 ± 9.22% for free vincristine, 41.01 ± 5.35% for vincristine liposomes, 52.66 ± 5.39% for vincristine plus dasatinib liposomes, and 66.00 ± 4.04% for functional vincristine plus dasatinib liposomes. These findings indicate that the functional vincristine plus dasatinib liposomes had the strongest overall anticancer efficacy among the different formulations.

The efficacy of VM channel elimination was evaluated using the periodic acid-Schiff (PAS)-CD34 dual staining assay, and the VM channels were stained in red in the tumor slices. After treatment, the rank of VM channel numbers in the tumor slices was physiological saline > free vincristine > vincristine liposomes> vincristine plus dasatinib liposomes > functional vincristine plus dasatinib liposomes (Figure 8B).

Figure 8C displays the induced apoptosis in the tumor tissue assessed using a terminal deoxynucleotidyl transferase-mediated dUTP nick end labeling (TUNEL) assay. The apoptotic cancer cells were displayed with green fluorescence under the confocal microscope. After treatment, functional vincristine plus dasatinib liposomes resulted in the most evident apoptosis of cancer cells compared with other controls.

In addition, the preliminary systemic toxicities after the treatments were evaluated via the body weight, blood examination and histopathological observation of major organs. Compared with physiological saline, there were no significant weight changes (Figure 8A2) or abnormalities in blood indicators (Supplementary Table S1) or major organs (heart, liver, spleen, lung, and kidney) (Figure 8D) after treatment with functional vincristine plus dasatinib liposomes.

### *In vivo* imaging in mice

**Figure 9:**
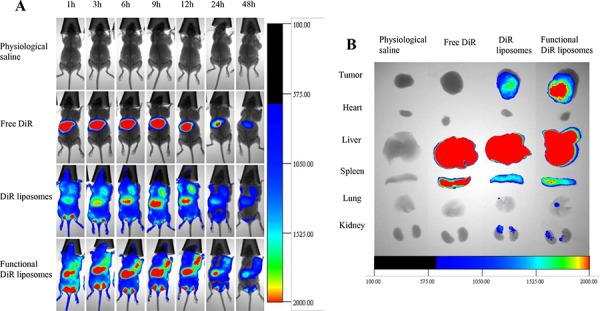
*In vivo* real-time imaging observation in nude mice after treatment with functional liposomes Notes: **A.** *In vivo* real-time imaging of the tumor-bearing nude mice. **B.** *Ex vivo* optical images of the tumor and normal tissues after the tumor-bearing mice sacrificed at 48 h.

Figure 9A depicts the real-time imaging and distribution of drug-loaded liposomes in tumor-bearing mice xenografted with MDA-MB-231 cells. Observations indicated that the functional DiR liposomes maintained longer circulation in the blood system and exhibited a higher accumulation in tumor tissues, for up to 48 h. In contrast, the fluorescent signal in tumor tissues gradually decreased at 12 h after treatment with DiR liposomes, whereas the fluorescent signal rapidly accumulated in the liver after treatment with free DiR.

Figure 9B illustrates the *ex vivo* optical images of tumor masses and major organs after the mice were sacrificed. Results showed that the fluorescent signals remained clearly visible in the tumor masses after treatment with functional DiR liposomes. In comparison, the fluorescent signals in the tumor masses were weakly visible after treatment with DiR liposomes and invisible after treatment with free DiR or physiological saline. In addition, strong fluorescent signals were identified in the livers and spleens after treatment with all DiR formulations.

## DISCUSSION

The residual aggressive cancer cells of TNBC can form the VM channels by self-transforming to support further growth of cancer tissue, leading to relapse and metastasis of the cancer. The VM channels are detected in clinical breast cancer specimens and associated with a poor 5-year survival rate [23–25]. Therefore, the functional vincristine plus dasatinib liposomes were developed for treatment of TNBC along with deletion of the VM channels.

In the functional liposomes, vincristine was encapsulated into the inner vesicle, and dasatinib was incorporated into the lipid bilayer of DSPE-PEG_2000_-c(RGDyK) modified liposomes. The liposomes exhibited good characterizations of small and well-distributed particle size, high encapsulation efficiencies (Table 1), smooth surface (Figure 2B2) and delayed drug release (Figure 2C). The particle size is suitable to avoid kidney filtration but allows to be accumulated into cancer tissues via EPR effects [26]. This delayed drug release is beneficial to prevent rapid leakage during circulation in the blood system and increase drug accumulation in cancer tissues.

TNBC cells and VM-capable cancer cells are drug-resistant, and the improvement of cellular uptake is the first step to enhance their efficacy. To address this issue, c(RGDyK) peptide was used as a targeting molecule for binding with the integrin receptor overexpressed on these cells. In evaluating cellular uptake (Figure 3A and 3B), the results demonstrate that the fluorescent intensity of functional coumarin liposomes is clearly stronger than the unmodified liposomes, which indicates the increased cellular uptake by cancer cells. Moreover, free coumarin also exhibits clear ingestion by cancer cells because of its direct contact permeation. The targeting effect is observed with a confocal microscope (Figure 3C). After incubation with functional liposomes, a bright yellow fluorescence was observed, indicating the targeting effect of functional liposomes with integrin β3 receptor on MDA-MB-231 cells.

In the inhibitory effects assay (Figure 4A), stronger inhibitory effects are observed after co-treatment of free vincristine with free dasatinib, indicating that dasatinib can enhance anticancer effects possibly by inducing apoptosis of cancer cells. Compared with other liposomes, functional vincristine plus dasatinib liposomes exhibit the strongest inhibitory effects on MDA-MB-231 cells (Figure 4B). This is attributed to the increased cellular uptake by the targeted action.

Apoptosis is a process of programmed cell death involved in cellular stress [27]. In this study, functional vincristine plus dasatinib liposomes exhibited the strongest inducing effects via the activation of caspases 8, 9, and 3 and the pro-apoptotic protein Bax or the suppression of the anti-apoptotic protein Mcl-1 (Figure 5A to 5C). The activation effect on caspase 8 and caspase 9 demonstrate the involvement of the death receptor signaling pathway and the mitochondrial signaling pathway during apoptosis, respectively [28–31]. As upstream initiators of apoptosis, activated caspases 8 and 9 initiate the activation of the downstream effector caspase 3, which leads to a cascade of apoptotic reactions in the cancer cells [32]. When being activated, pro-apoptotic protein Bax forms dimers and permeabilizes the mitochondrial outer membrane, thereby inducing apoptosis [33, 34]. In contrast, the anti-apoptotic protein Mcl-1 neutralizes the activity of pro-apoptotic proteins, and ultimately retard apoptotic reactions [35]. Under the induction of functional vincristine plus dasatinib liposomes, the balance slants to promote apoptotic reactions by increasing Bax activity and suppressing Mcl-1 activity.

Appropriate level of ROS plays important role in keeping redox balance and cell proliferation under physiological conditions [36]. However, under oxidative stress conditions, excess ROS exhibit a cytotoxic nature. Excessive ROS levels in MDA-MB-231 cells were produced by treatment with functional vincristine plus dasatinib liposomes (Figure 5D), and the generated ROS could be involved in mitochondrial membrane damage and cytochrome C release, which induce apoptosis as well [37]. Besides, the greater ROS levels also cause acute injury by necrosis, which thus increases the overall cytotoxicity in the cancer cells.

Under hypoxic conditions, phenotype-transformed invasive cancer cells can form VM channels after treatment [38]. The functional vincristine plus dasatinib liposomes displayed the strongest destructive effect on VM channels (Figure 6A) through the increased cellular uptake, the induced apoptosis and the inhibited marker molecules. During VM channel formation, VE-Cad transcription is increased, and tyrosine kinase receptor A2 (EphA2) is subsequently re-localized to the cell membrane and phosphorylated. The activated EphA2 proteins promote the localization of FAK to new focal adhesion sites and subsequently activate PI3K, which thus triggers cell migration and VM channel formation. In addition, the activated VE-Cad and EphA2 proteins also directly activate PI3K, which then promotes the MMP precursor to form MMPs, ultimately promoting the cancer cell binding to form VM channels. After treatments with functional vincristine plus dasatinib liposomes, the expressions of these protein indicators were significantly decreased (Figure 6B to 6F), suggesting a destroying effect on the VM channels, hence being beneficial for eradicating the TNBC.

Multicellular TNBC tumor spheroids were used to simulate a solid tumor environment [39]. Results demonstrated that the functional coumarin liposomes show a strong penetrating ability (Figure 7A), and the functional vincristine plus dasatinib liposomes exhibit a strong destructing efficacy (Figure 7B and 7C). These results indicated the roles of functional vincristine plus dasatinib liposomes in potentially treating refractory solid TNBC with a strong penetrating ability and a destructing efficacy.

The *in vivo* study was evaluated on tumor-bearing mice from several aspects: anticancer efficacy, induction of apoptosis, VM channels elimination and safety evaluation. The functional vincristine plus dasatinib liposomes exhibited the strongest overall anticancer efficacy (Figure 8A1), elimination of VM channels (Figure 8B) and induction of apoptosis (Figure 8C) in tumor tissues. The enhanced efficacy could be explained as follows: the pegylated liposomes showed a long circulatory effect (Figure 9) by escaping from the rapid clearance by RES in blood circulation [40]; the suitable particle size of the liposomes allows for more accumulation in tumor tissue (Figure 9) by EPR effects; functional liposomes could improve the cytotoxic effects on cancer cells by the increased cellular uptake; and the combination therapy of vincristine and dasatinib enhances the apoptosis and eliminate the VM channels by inhibiting VM indicators.

## MATERIALS AND METHODS

### Materials and reagents

DSPE-PEG_2000_ and DSPE-PEG_2000_-NHS were obtained from the NOF Corporation (Tokyo, Japan). The cyclic RGD peptide (c(RGDyK)) was synthesized by GL Biochem Co., Ltd. (Shanghai, China). Vincristine sulfate and dasatinib were supplied by Nanjing Tianzun Zezhong Chemicals, Co., Ltd. (Nanjing, China). Other chemicals were analytical or high performance liquid chromatography grade.

### Cancer cells and animal experimentation

Human breast cancer MDA-MB-231 cells were purchased from the Institute of Basic Medical Science, Chinese Academy of Medical Science (Beijing, China). Cells were grown in Leibovitz's L15 medium (Macgene, Beijing, China) supplemented with 10% fetal bovine serum (FBS, Gibco, Billings, USA) in a 37°C humidified incubator. Female BALB/c nude mice (initial weight of 16–18 g) were obtained from the Peking University Experimental Animal Center (Beijing, China). All animal experimental procedures were performed with the approval of the Institutional Authority for Laboratory Animal Care of Peking University according to the guidelines.

### Synthesis of targeting molecules

DSPE-PEG_2000_-c(RGDyK) conjugate was synthesized via the following procedures. Briefly, c(RGDyK) peptide and DSPE-PEG_2000_-NHS were dissolved at a ratio of 2:1 (mol/mol) in anhydrous dimethylformamide, and the pH of the reaction solution was adjusted to 9.0 using N-methyl morphine. The solution was then continuously stirred at room temperature for 24 h. The reaction mixture was subsequently transferred into dialysis tubing (cut-off MW, 3000 Da) and dialyzed against deionized water for 48 h. The resultant was then lyophilized and stored at −20°C. The product was confirmed using a MALDI-TOF-MS (Bruker Daltonics, Germany).

### Preparation and characterization of liposomes

Liposomes were prepared using the film dispersion method, and drugs were loaded using the ammonium sulfate gradient loading method as previously reported [41]. Briefly, egg phosphatidylcholine, cholesterol, DSPE-PEG_2000_, DSPE-PEG_2000_-c(RGDyK) conjugate and dasatinib were dissolved in chloroform and methanol (3:1, v/v) at a ratio of 66:26:2.5:3:3.5 (mol/mol) in a pear-shaped bottle. The solvent was removed by a rotary vacuum evaporator, and the lipid film was hydrated with 250 mM ammonium sulfate by water-bath sonication for 5 min. The suspensions were subsequently treated using an ultrasonic cell disruptor for 10 min and successively extruded through polycarbonate membranes with pore sizes of 400 and 200 nm, 3 times each. The suspensions obtained were dialyzed (cut-off MW, 12,000–14,000 Da) in Hepes buffered saline (25 mM Hepes/150 mM NaCl) for 24 h and incubated with vincristine solution in a water bath at 40°C with continual shaking for 30 min (lipids: drug = 20:1, w/w). Then, functional vincristine plus dasatinib liposomes were obtained. Blank functional liposomes, vincristine plus dasatinib liposomes and vincristine liposomes were prepared using the same procedures by excluding the addition of dasatinib or DSPE-PEG_2000_-c(RGDyK) conjugate or DSPE-PEG_2000_-c(RGDyK) conjugate and dasatinib, respectively, during the film formation process. Moreover, using fluorescent probes to evaluate the distribution *in vivo*, four types of liposomes were similarly prepared, including coumarin liposomes, functional coumarin liposomes (lipids: coumarin = 500:1, w/w), DiR liposomes and functional DiR liposomes (lipids: DiR = 200:1, w/w).

The particle sizes, PDI and zeta potential values were measured using a Nano Series Zen 4003 Zetasizer (Malvern Instruments Ltd, Malvern, UK). The morphology of the liposomes was observed using an AFM (SPI3800N series SPA-400, Tokyo, Japan). The *in vitro* release of vincristine or dasatinib from the liposomes was achieved by dialysis against phosphate buffered saline (PBS, pH 7.4) that contained 10% FBS at 37°C.

### Cellular uptake by TNBC cells and targeting effect

Coumarin was used as a fluorescent probe to determine the cellular uptake. To evaluate the cellular uptake, MDA-MB-231 cells were seeded at a density of 4 × 10^5^ cells/well in 6-well culture plates. After 24 h, the cells were treated with free coumarin, coumarin liposomes and functional coumarin liposomes at a final concentration of 0.5 μM coumarin for another 4 h. Culture medium was used as a blank control. After incubation, the cells were collected to measure the fluorescence intensity using a flow cytometer (Becton Dickinson, USA) according to the manufacturer's instructions. Each assay was repeated in triplicate.

To evaluate the targeting effect, MDA-MB-231 cells were seeded into chambered cover slides at a density of 2 × 10^5^ cells/dish. After 24 h incubation, the cells were treated with free coumarin, coumarin liposomes and functional coumarin liposomes at a final concentration of 1 μM coumarin for another 2 h. Culture medium was used as a blank control. After incubation, the cells were fixed with 4% paraformaldehyde for 10 min, and then blocked with 10% goat serum that contained 0.3 M glycine for 2 h at room temperature. After being washed by PBS (pH 7.4), MDA-MB-231 cells were incubated with anti-integrin β3 antibody (1:100 dilution, Abcam) at 4°C overnight, followed by incubation with Alexa Fluor 647-conjugated (1:500 dilution, Abcam) at room temperature for 2 h. After being washed by PBS, nuclei were stained with Hoechst 33342 (2 μg/mL) for 10 min. Finally, the cells were imaged and analyzed using a confocal laser scanning fluorescent microscopy (Leica, Heidelberg, Germany).

### Inhibitory effects and induction of apoptosis on TNBC cells

To compare the inhibitory effects of the different formulations, MDA-MB-231 cells were seeded at a density of 7000 cells/well in 96-well culture plates and cultured for 24 h. The cells were subsequently treated with serial concentrations of the drug formulations. The final concentration of vincristine ranged between 0–1 μM, whereas the concentration of dasatinib was in the range of 0.1–1.0 μM. Culture medium was used as a blank control. After treatment for 48 h, the inhibitory effects were determined using a sulforhodamine-B staining assay [42]. Survival rates were calculated using the following formula: survival (%) = (A_540 nm_ for treated cells/A_540 nm_ for control cells) × 100%, where A_540 nm_ is the absorbance at 540 nm as measured by a microplate reader (Infinite F50, Tecan Group Ltd., Shanghai, China).

Apoptosis was identified using a fluorescein annexin V staining kit (Biosea Biotechnology Co., Ltd, Beijing, China). MDA-MB-231 cells were seeded at a density of 4 × 10^5^ cells/well in 6-well culture plates for 24 h at 37°C. After 24 h incubation, the cells were treated with varying drug formulations at a concentration of 0.1 μM vincristine or dasatinib. Culture medium was used as a blank control. After incubation for 6 h, the cells were handled according to the manufacturer's protocol and assessed via flow cytometry. Each assay was repeated in triplicate.

### Apoptotic signaling pathways of TNBC cells

MDA-MB-231 cells were seeded in 96-well plates and incubated for 24 h, followed by treatment with varying drug formulations at a concentration of 0.1 μM vincristine or dasatinib. Culture medium was added as a blank control. After incubation for 6 h, the cells were fixed with 4% formaldehyde for 15 min, permeabilized with 0.5% Triton X-100 for 15 min and blocked with 10% goat serum that contained 0.3 M glycine for 2 h at room temperature. The cells were subsequently incubated with the primary antibody (Sangon, China) at 4°C overnight, followed by incubation with the secondary antibody conjugated with Alexafluor-488 (OriGene, China) at room temperature for 2 h. Both primary and secondary antibodies were properly diluted according to the instructions. Nuclei were stained with Hoechst 33342 (2 μg/mL) for 10 min at room temperature. The fluorescence intensity of each well was measured using the Operetta high content screening system and calculated with the Columbus system.

To evaluate the ROS-related apoptotic pathway, MDA-MB-231 cells were seeded in 6-well plates at a density of 3 × 10^5^ cells/well and incubated for 24 h. The formulation administrations were the same as previously described. After 6 h incubation with varying formulations, the cells were stained with 1 μM DCFH-DA (Biotime, China) for 10 min. The cells were then washed, harvested and re-suspended in PBS and determined immediately via flow cytometry.

### Destruction of VM channels of TNBC cells

A matrigel-based tube formation assay was used to assess the destructive effect on the VM channels of MDA-MB-231 cells [43]. Briefly, culture plates (96-well) were coated with matrigel (50 μL/well) and allowed to polymerize at 37°C for 30 min. MDA-MB-231 cells were collected and resuspended with serum-free culture medium at a density of 1 × 10^4^ cells/well and subsequently seeded in wells that contained varying formulations at a concentration of 0.01 μM vincristine or dasatinib. Blank medium was used as a control. After incubation for 10 h, each well was photographed and analyzed using an EVOS microscope.

The expression levels of six proteins (VE-Cad, FAK, PI3K, EphA2, MMP-2, and MMP-9) in MDA-MB-231 cells were determined using enzyme-linked immunosorbent assay kits (Cusabio Biotech Co. Ltd., Beijing, China). Briefly, cells were cultured to 80% confluence and then treated with varying formulations for 12 h. The final concentration of vincristine or dasatinib was 0.1 μM. Culture medium was used as a blank control. After incubation, the cells were harvested and lysed. The cell lysates were analyzed using a microplate reader according to the manufacturer's instructions for the kits.

### Destruction of TNBC tumor spheroids

Multicellular tumor spheroids of MDA-MB-231 cells were grown *in vitro* using the liquid overlay system [44]. Briefly, agarose was added to serum-free culture medium and heated to 80°C for 30 min to form a 2% (w/v) solution. Each well of a 96-well culture plate was coated with 50 μL agarose solution. After cooling to ambient temperature, MDA-MB-231 cells were seeded at a density of 1 × 10^3^ cells/well with 100 μL growth medium. The culture plates were gently shaken for 5 min and incubated for 48 h to form tumor spheroids.

To monitor the penetration ability, coumarin was used as a fluorescent probe. The MDA-MB-231 tumor spheroids were treated with free coumarin, coumarin liposomes, and functional coumarin liposomes for 12 h, respectively. The final concentration of coumarin was 10.0 μM. After incubation, the spheroids were washed with PBS and subsequently scanned at different layers from the top of the spheroids to the inside using the confocal laser scanning fluorescent microscope.

To evaluate the destructive effects of different formulations, MDA-MB-231 tumor spheroids were collected in 6-well culture plates and then treated with varying formulations at a concentration of 1 μM vincristine or dasatinib. Culture medium was used as a blank control. After incubation for 48 h, the spheroids were fixed by 2.5% glutaraldehyde for 60 min, rinsed three times with PBS, dehydrated and embedded. The spheroids were subsequently observed under a scanning electron microscope (SEM, JSM-5600 LV, JEOL, Japan).

After a tumor spheroid was formed in a well, varying formulations were added at a concentration of 1 μM vincristine or dasatinib. Culture medium was used as a blank control. The inhibitory effect was evaluated via the measurement of the tumor spheroid under an inverted microscope. Briefly, the major (d_max_) and minor (d_min_) diameters of each spheroid were measured, and the spheroid volume was calculated as previously described by the following formula: V = (π × d_max_ × d_min_) / 6 [45]. The tumor spheroid volume ratio was subsequently calculated using the formula R = (V_day i_ / V_day 0_) × 100%, where V_day i_ is the tumor spheroid volume at the ith day (day after drug treatments), and V_day 0_ is the tumor spheroid volume prior to treatment.

### Anticancer efficacy

Approximately 1 × 10^7^ MDA-MB-231 cells were subcutaneously injected into the right armpits of BALB/c female nude mice. Following growth to a volume of approximately 1000 mm^3^, the tumors were extracted, cut into pieces of approximately 2 × 2 × 2 mm^3^, and then seeded into the right armpits of fresh nude mice with a tumor inoculation needle. When the tumors reached approximately 100 mm^3^ in volume, the mice were randomly divided into five treatment groups (*n* = 6) and treated with physiological saline, free vincristine, vincristine liposomes, vincristine plus dasatinib liposomes, or functional vincristine plus dasatinib liposomes via the tail vein at days 12, 14, 16, 18, 20 and 22 after inoculation. The dosage of vincristine was 1 mg/kg (vincristine: dasatinib = 1:1, mol/mol). The mice were weighed, and the tumors were measured with calipers. The tumor volumes (V) were calculated with the formula V = (length × width^2^) / 2 (mm^3^), and the tumor volume ratios were evaluated with the formula R (%) = V_day i_ / V _day 11_× 100%, where V_day i_ is the tumor volume at day i, and V_day 11_ is the tumor volume at day 11.

A routine blood analysis of the mice was conducted using an MEK-6318K Hematology Analyzer (Nihon Kohden, Japan) prior to euthanasia. The mice were subsequently euthanized at day 25 via cervical dislocation. The tumor masses were carefully isolated to prepare cryosections for the TUNEL assay using an in situ apoptosis detection kit (KeyGen Biotechnology Co., Ltd, Nanjing, China). The FITC-labeled TUNEL-positive cells were imaged via the confocal laser scanning fluorescent microscopy. To evaluate the *in vivo* destructive effect on VM channels, CD34 endothelial marker PAS dual staining was used [46]. Briefly, the assay was performed on the tumor slices using a CD34 antibody (1:300 dilution, Santa Cruz Biotechnology, USA), Peroxidase Kit (Santa Cruz Biotechnology, USA) and PAS staining according to the manufacturer's protocol and ultimately photographed using an EVOS microscope. Other organs were removed to create paraffin sections for hematoxylin and eosin staining for safety examination.

### *In vivo* imaging in mice

Non-invasive optical imaging systems were used to observe the real-time distribution and tumor accumulation ability of DiR-loaded liposomes in MDA-MB-231 xenografts. When the tumors reached approximately 500 mm^3^ in volume, the mice were randomly divided into four groups (3 mice per group). The mice were subsequently administered physiological saline, free DiR, DiR liposomes, or functional DiR liposomes via the tail vein. The mice were then anesthetized with isoflurane and scanned at 1, 3, 6, 9, 12, 24 and 48 h using a Kodak multimodal imaging system (Carestream Health, Inc., USA). To further observe the distribution status, the mice were immediately euthanized to collect the tumor masses and major organs. The fluorescence signal intensities in different tissues were measured.

### Statistical analysis

Data are presented as the means ± standard deviations (SDs). One-way analysis of variance was used to determine the significance among the groups, after which post-hoc tests using a Bonferroni correction were used for multiple comparisons between individual groups. A *p* value < 0.05 was considered significant.

## CONCLUSIONS

In this study, a type of nanostructured functional vincristine plus dasatinib liposomes was developed via modification with the targeting molecule DSPE-PEG_2000_-c(RGDyK). The liposomes exhibited a significant efficacy in TNBC MDA-MB-231 cells *in vitro* and in TNBC-bearing nude mice. The action mechanism involved the following aspects: (i) the pegylated liposomes and the nanostructured particle size resulted in prolonged circulation in the blood and more drug accumulation in the tumor tissue; (ii) the targeting molecule promoted cellular drug uptake; (iii) the induction of apoptosis potentiated the overall anticancer efficacy via activation of capases (8,9, and 3), increased expression of Bax, decreased expression of Mcl-1, and generation of ROS; and (iv) the destructive effect on the VM channels prevented the residual cancer cells from the relapse after treatment via inhibition of VM channel indicators, which consisted of VE-Cad, FAK, PI3K, MMP-2, and MMP-9. It is concluded that the functional vincristine plus dasatinib liposomes may provide a potential strategy for treatment of TNBC along with elimination of VM channels.

## SUPPLEMENTARY TABLE


