# Supplementary material for: Application of functional vincristine plus dasatinib liposomes to deletion of vasculogenic mimicry channels in triple-negative breast cancer
Source: Oncotarget. 2015 Sep 28;6(34):36625–42. doi: 10.18632/oncotarget.5382 (PMC4742200; doi:10.18632/oncotarget.5382)
Supplement: Supplementary file 1 [file oncotarget-06-36625-s001.pdf]

SUPPLEMENTARY TABLE

Supplementary Table S1: Blood examination of tumor-bearing nude mice after treatment with varying formulations at day 22 after inoculation

Assay	Physiological saline	Vincristine liposomes	Vincristine plus dasatinib liposomes	Functional vincristine plus dasatinib liposomes	Free vincristine
WBC[a)] (10^9/L)	5.57 ± 1.51	6.20 ± 1.35	7.80 ± 2.02	5.57 ± 1.16	3.53 ± 0.46*
RBC[b)] (10^{12}/L)	7.75 ± 0.46	7.13 ± 0.77	7.81 ± 1.07	8.30 ± 1.02	8.59 ± 2.96
MCHC[c)] (g/L)	304.67 ± 1.53	299.00 ± 20.66	298.33 ± 2.31	291.00 ± 12.53	299.67 ± 26.50
RDW[d)] (%)	14.10 ± 0.92	13.30 ± 0.50	13.33 ± 0.45	13.23 ± 0.12	13.67 ± 1.98
LYM[e)] (10^9/L)	0.93 ± 0.84	2.77 ± 2.25	1.13 ± 0.45	1.77 ± 0.15	0.50 ± 0.20
MID[f)] (10^9/L)	1.30 ± 0.78	1.33 ± 0.76	1.03 ± 0.42	0.90 ± 0.10	0.50 ± 0.30

[a)]White blood cells; [b)]Red blood cells; [c)]Mean corpuscular hemoglobin concentration; [d)]Red cell distribution width; [e)]Lymphocytes; [f)]Intermediate cell number. Data are presented as the mean ± SD (n = 3). *$p < 0.05$, vs. physiological saline.
